# Comprehensive Comparison of Features of Robotic Surgery Systems in Abdominal Surgeries: A Narrative Review Study

**DOI:** 10.1002/hsr2.71894

**Published:** 2026-02-23

**Authors:** Mashallah Torabi, Mehrnaz Aghanouri, Fatemeh Hadavandsiri, Maryam Goodarzi

**Affiliations:** ^1^ Medical Image and Signal Processing Research Center, School of Advanced Technologies in Medicine Isfahan University of Medical Sciences Isfahan Iran; ^2^ Department of Medical Physics and Biomedical Engineering, School of Medicine Tehran University of Medical Sciences Tehran Iran; ^3^ Public Relation Tehran University of Medical Sciences Tehran Iran; ^4^ Department of Epidemiology, School of Public Health and Safety Shahid Beheshti University of Medical Sciences Tehran Iran; ^5^ Management of Technology Tehran University of Medical Sciences Tehran Iran

**Keywords:** robotic‐assisted surgery, robotic surgery, surgical systems

## Abstract

**Background and Aims:**

Over the past two decades, robotic‐assisted abdominal surgery has revolutionized the field of abdominal surgery. This narrative review aims to provide a comprehensive and current analysis of the latest robotic surgery systems employed in abdominal procedures, emphasizing their design, capabilities, and unique features.

**Methods:**

We conducted a systematic search using PubMed, Google, and manufacturer websites, utilizing keywords such as robotic surgery, robotic‐assisted surgery, and specific robotic systems including da Vinci, Hugo, Versius, Flex, Senhance, Revo‐I, MicroHand S, Hinotori, Avatera, Bitrack, ENOS (SPORT), MIRA, Mantra, Medrobotics Flex, Dexter, Saroa, Toumai, SHURUI, and Sina_*flex*_.

**Results:**

Our review identified and analyzed 19 robotic systems from various countries, including the United States, Japan, South Korea, Iran, and China. This review not only highlights the features of these emerging robotic surgery systems but also delves into less‐studied systems, such as the Saroa and Sina_*flex*_ robotic surgery systems, thereby addressing a gap in the current literature.

**Conclusion:**

Robotic‐assisted abdominal surgery continues to advance, offering greater precision and reduced invasiveness. Although high costs remain a challenge, ongoing technological progress and the introduction of new systems are improving both accessibility and performance. With the growing integration of artificial intelligence, robotic surgery is expected to become even more efficient, ultimately supporting surgeons and enhancing patient outcomes.

## 1 Introduction

In the early 2000s, the revolution of robotic‐assisted surgery significantly improved the management of urological conditions, becoming the preferred surgical approach in many cases [1]. As a form of minimally invasive surgery (MIS), robotic systems use small instruments inserted through tiny incisions, controlled by the surgeon from a surgeon console to manipulate patient‐side robotic arms. These systems translate the surgeon's hand, wrist, and finger movements into scaled, precise actions, offering superior accuracy, flexibility, and control compared with conventional techniques [2].

Robotic‐assisted surgery has addressed key limitations of laparoscopy and expanded MIS indications to more delicate and complex procedures. This advancement is driven by magnified three‐dimensional (3D) high‐definition (HD) visualization, enhanced dexterity, tremor filtration, and highly precise movements. Compared with open or laparoscopic approaches, robotic surgery is associated with faster recovery [3], shorter hospital stay [4], and reduced blood loss and transfusion rates [5].

For nearly two decades, the da Vinci surgical system (Intuitive Surgical, Sunnyvale, CA, USA) has been the leading platform for robot‐assisted laparoscopic surgery, maintaining market dominance since its launch in 1999 and FDA approval in 2000 [6]. Over the past decades, several new robotic surgical systems have emerged and entered clinical practice [5], marking an exciting era in which increased competition promotes technological evolution and cost reduction.

Despite its widespread use, the da Vinci system has been criticized for several technical and practical limitations. These include limited communication between surgeon and operating team due to the isolated console design, the absence of tactile feedback, difficulty in arm positioning that restricts surgical access, and the large size that occupies substantial operating room space [5, 7].

This article reviews the advantages and strengths of surgical robots developed over the years, comparing their characteristics to provide a comprehensive overview of robotic platforms worldwide. The review aims to help audiences in different regions better understand available systems and make informed decisions based on their own resources.

## 2 Method and Materials

To obtain comprehensive information on robotic‐assisted surgery systems, we performed a comprehensive search across multiple sources, including PubMed, Google, and manufacturer websites. The Google search was conducted globally to ensure broad data coverage. Keywords included “robotic surgery,” “robotic‐assisted surgery,” and the names of specific robotic systems (e.g., da Vinci, Hugo, Versius, Senhance, Revo‐I, MicroHand S, Hinotori, Avatera, Bitrack, ENOS (SPORT), MIRA, Mantra, Medrobotics Flex, Dexter, Saroa, Toumai, SHURUI, and Sinaflex). This strategy enabled the identification of the latest developments and features of robotic systems from various countries, including the United States, Japan, South Korea, China, and Iran. We reviewed robots capable of performing abdominal surgeries, including platforms certified for all abdominal procedures, those approved for specific procedures, and systems currently undergoing clinical trials and pending certification. The research was conducted collaboratively by two authors, F. H. and M. A., who contributed equally to study design, literature search, reviewing and integrating the available information, and manuscript preparation. As this study is a narrative review that did not involve the collection, analysis, or reporting of data from human participants, no ethical approval or informed consent was required in accordance with applicable institutional and international guidelines.

## 3 Results

Each of the robotic surgery systems found by the explained method of search is briefly introduced as follows, and its features are depicted. In Table 1, some key features of robotic surgery systems are selected and compared for each available system.

**Table 1:** Key features of the robotic surgery systems, either commercialized (received national or international clearance) or in progress.

Robotic platform	Approvals	Country	Installation locations	Robotic surgical systems			Patient cart	Surgeon console	Control (Handles type)	Number of arms	Camera diameter (mm)	Instrument use	Instrument diameter	Availability
				Multiple port	Single port	Natural orifice								
da Vinci Xi	FDA/2014 CE Mark/2014	USA	Over 6000 systems all around the world	*			Single	Closed	Finger loops	4	5–8	12–18 times	8–12 mm	Commercial
da Vinci SP	FDA/2018 CE/2024 MHLW/2022^a^	USA	United States, Korea, and Japan		*		Single	Closed	Finger loops	1 (3 instruments and 1 camera)	12*10	25–150 times	Port: 25 mm Instrument: 6–7 mm	Commercial
Versius	CE Mark/2019 TGA/2019^b^	UK	Over 160 Versius installations around the world	*			Multiple	Open (3D glasses)	Pistol type	3–6	10	Reusable	5 mm	Commercial
Senhance	FDA/2017 CE‐mark TGA	USA	In several hospitals around the world	*			Multiple	Open (3D glasses)	Laparoscopic handles	2–4	10	Reusable	3–5 mm	Commercial
Hinotori	MHLW/2020 Singapore/2023	Japan	—	*			Single	Semi‐closed	Finger loops	4	N/A	10 times	N/A	Commercial
Revo‐I	Korean Ministry of Food and Drug granted/2017 TGA	Korea	USA, Asia, including Uzbekistan, Japan, Europe and other countries	*			Single	Closed	Finger loops	4	10	20 times	7.4 mm	Commercial
Hugo	CE‐mark/2021	USA	Several countries all around the world	*			Multiple	Open	Pistol type	3–4	11	Reusables (Some disposables)	8 mm	Commercial
Avatera	CE‐mark/2019	Germany	Germany, Denmark Greece, France, Hungary	*			Single	Semi‐closed	Finger loops	4	10	Single‐use	5 mm	Commercial
Sina_*flex*_	In progress 2024	Iran	Iran, Indonesia	*			Multiple	Open	Pistol type	3–4	10	Single‐use	5 mm	In progress
Dexter	CE Mark/2020	Switzerland	N/A	*			Multiple	Open	Finger loops	3	12 mm camera trocar	Single‐use	8 mm	Commercial
Bitrack	In progress 2024	Spain	N/A	*			Single	Open	Finger loops	4	3D‐HD screen	Single‐use	8 mm	In progress
Flex	FDA/2017 CE Mark/2016	USA	N/A			*	Single	Open	Joystick	1	N/A	Single‐use	Port: 5–10 mm Instrument: 2 or 3.5	Commercial
Mantra	CDSCO, India^c^	India	India, Sri Lanka, Nepal, Bangladesh, and Indonesia	*			Multiple	Open	Mini joystick	3–5	N/A	N/A	9 mm	Commercial
MicroHand S	NMPA approval/2021^d^	China	N/A	*			Single	Open	Finger loops	3	N/A	Reusable	8 mm	Commercial
Saroa	Manufacturing and marketing approval in Japan in May 2023	Japan	N/A	*			Single	Open	N/A	3	N/A	N/A	N/A	In progress
Toumai or Microport	NMPA/2022	China	N/A	*			Single	closed	Finger loops	4	N/A	N/A	8.5 mm	Commercial
MIRA	Completed IDE for bowel resections^e^	USA	N/A		*		Single	Open	Pistol type	2	N/A	Reusable	N/A	In progress
ENOS (SPORT)	In progress 2024	Canada	N/A		*		Single	Open	Pistol type	Up to 6	N/A	Reusable	N/A	In progress
SHURUI SP robot	NMPA for clinical use/2023	China	N/A		*		Single	Semi‐closed	Finger loop	3 instruments and 1 camera	10 mm	Reusable	8 mm	In progress

^a^ Japanese Ministry of Health.

^b^ Therapeutic Goods Administration (Australia).

^c^ The Central Drugs Standard Control Organization (India).

^d^ National Medical Products Administration (China).

^e^ Investigational Device Exemption (USA).

### 3.1 Pre da Vinci Surgical System Era

- Robotic surgery originated from NASA and US defense research in the 1970s to enable remote‐controlled operations in hazardous environments [8].
- **PUMA 560** was the first surgical robot used for a neurosurgical biopsy in 1985, followed by **ProBot** for prostatectomies in 1988 [9].
- **Computer Motion** was the leading supplier of surgical robots, with products such as **AESOP** and **ZEUS** in the 1989s, which were used for the first time in 1998 at the Cleveland Clinic [10, 11].

### 3.2 Multiple Port Surgical Robotic Systems

#### 3.2.1 da Vinci

Intuitive Surgical introduced the first‐generation da Vinci surgical robot in 1999, receiving FDA approval in 2000. This system comprised a patient‐side robotic arm, a surgeon console with a stereoscopic viewer, and a 3D endoscopic imaging system. The robotic arms offered high flexibility and precision, while the stereoscopic viewer provided a clear, realistic surgical field. In 2002, the system was upgraded to four arms, and the surgeon console included handles, pedals, and a device to control arm movements and energy instruments [12].

After a 3‐year legal dispute, Computer Motion and Intuitive Surgical merged in 2003, discontinuing the ZEUS system and incorporating its features into future developments [13]. To date, six da Vinci models have been introduced: S and Si Systems (2003, 2009), Xi and X Systems (2014, 2017), the Single‐Port (SP) System (2018), and da Vinci 5 (2024) [8, 13].

The Si platform (third generation) added dual console, FireFly fluorescence imaging, and an endowrist stapler but retained limitations, including large arms, vertical column, specific scope arm, and difficulty in multiquadrant surgery [12]. The Xi platform (fourth generation) improved instrumentation, vision, cart design, table motion, and setup automation. It addressed prior limitations with smaller, more flexible arms, a lateral column, universal scope arm, and easier docking for multiquadrant surgery. The Xi system features a redesigned patient cart, single‐fin attachment, flex joint architecture, and an 8‐mm 3D‐HD endoscope that is bright, high‐resolution, invertible from the console, and does not require draping or calibration. Additional improvements include FireFly imaging, enhanced instruments, energy and stapling devices, reusable ports, robotic suction/irrigation, clip application, vessel sealing, single‐site technology, and a wristed needle driver for suturing [12].

#### 3.2.2 Versius

The Versius surgical system, developed in the United Kingdom, aims to enhance accessibility and versatility in minimal access surgery [5]. It received European CE Mark approval in 2019 [14]. The system features an adaptable open console suitable for sitting or standing and up to six modular robotic arms [15, 16]. The lightweight console provides 3D‐HD vision, joystick handles, and haptic feedback. The robotic arms offer 7 degrees of freedom (DoF), mimicking human arm movements [17, 18], while the camera is controlled via the surgeon's head movement. Fully wristed, 5‐mm instruments are reusable.

Versius has been evaluated in preclinical and clinical settings, demonstrating feasibility and safety across urology, gynecology, and general surgery procedures [19, 20]. By November 2022, it had been installed in over 100 hospitals and had performed more than 5000 surgeries [21].

#### 3.2.3 Senhance

The Senhance surgical system, developed by an Italian company and later acquired by Asensus Surgical (USA), received European approval in 2014 and FDA approval in 2017. It features up to four modular robotic arms without the need for port docking. Instruments are 3‐ or 5‐mm laparoscopic tools with 6 DoF and are fully reusable. The surgeon operates from an open console with ergonomic seating, 3D‐HD visualization, polarized glasses, and haptic feedback. Eye‐tracking controls the camera, adjusting the field of view. Senhance is compatible with traditional laparoscopy and aims to reduce costs through reusable instruments [22, 23]. It has been applied in various procedures, including urology, colorectal, and abdominal surgeries, primarily in Europe.

#### 3.2.4 Hinotori

The Hinotori robotic system, developed by Medicaroid Corporation in Kobe, Japan, received regulatory approval from the Japanese Ministry of Health, Labor and Welfare (MHLW) in August 2020 [24]. It is the first made‐in‐Japan robotic platform, initially intended for urological surgeries, particularly prostate cancer. The system comprises a surgeon cockpit, operative unit, and vision unit. The operative unit has four multi‐jointed robotic arms with eight axes of movement [25] and features vibration filtering and a docking‐free design to reduce interference with the bedside assistant [5]. The semi‐closed surgeon console allows ergonomic sitting and control of wristed instruments via loop‐like handles [26]. Instruments are reusable up to 10 times, and the 4K vision unit enables 3D microscopic tissue visualization.

Early human trials, including radical prostatectomy and robotic‐assisted partial nephrectomy, reported median console times and adverse events. To evaluate the effectiveness of the docking‐free design on clinical outcomes, a study was conducted on patients who underwent radical prostatectomy using either the Hinotori or da Vinci Xi robotic systems. The results demonstrated a reduction in postoperative pain among patients operated on with the Hinotori system compared to those treated with the da Vinci Xi [27]. The Hinotori system is expected to enter international markets within 2–3 years, pending regulatory approvals [25].

#### 3.2.5 Revo‐I

The Revo‐I surgical system, developed by Meere Company Inc. in South Korea, received approval from the Korean Ministry of Food and Drug Safety in 2017 [28]. It is a master‐slave platform featuring a closed ergonomic console, a four‐arm patient cart, and an HD vision system. The system provides warnings when excessive force is applied and uses a 10‐mm 3D endoscope with 7.4‐mm fully wristed instruments offering 7 DoF, reusable up to 20 times [29].

Revo‐I has been employed in procedures including prostatectomy, fallopian tube reconstruction, cholecystectomy, and partial nephrectomy. Clinical data indicate a shorter hospital stay compared with da Vinci Si, although operative time is longer. The system is more affordable, reducing instrument and maintenance costs by approximately 42% [30].

#### 3.2.6 Hugo

The Hugo RAS system (Medtronic, Minneapolis, MN, USA) is a versatile robotic platform with multiple ports, featuring an open console, independent arm carts, and advanced controllers. First used clinically in 2021 in Chile [31], it received CE mark approval for gynecological and urological procedures the same year, but is still awaiting FDA approval. Similar to Senhance, Hugo provides 3D visualization with dedicated glasses, uses an 11‐mm camera port and 8‐mm instrument ports, and offers a console with head‐tracking, haptic feedback, ergonomic seating, and arm rest support. Instruments are wristed and reusable [18, 32, 33].

Hugo has been employed in recent clinical studies, including robot‐assisted radical prostatectomy, reporting no intraoperative complications or technical failures [34, 35]. However, these studies were small, and larger multi‐center trials are anticipated [34, 35, 36].

#### 3.2.7 Avatera

The Avatera system, developed in Germany, received European approval in November 2019. Its patient cart has four robotic arms: three for 5‐mm fully wristed instruments with 7 DoF (single‐use), and one for a 10‐mm camera. The surgeon operates from a compact, ergonomically designed console with adjustable seating. A 3D full HD view is provided via a microscope‐like device that does not cover the surgeon's head, allowing better team communication. Instruments are controlled through loop‐shaped handles [37].

#### 3.2.8 Sina_*flex*_

The Sina_*flex*_ Robotic Telesurgery System, developed by Sina Robotics and Medical Innovators Co., Tehran, Iran, in late 2018, is designed for surgeries in the pelvic, abdominal, and thoracic regions via internet or other communication channels [38, 39]. The system features a reconfigurable console that allows the surgeon to sit or stand for optimal ergonomics, and modular robot placement on one or both sides of the surgical bed. Patient‐side robots measure tool‐tissue interaction forces, including instrument jaw pinch forces, and transmit haptic feedback to the surgeon through the surgeon console. Using 5‐mm disposable, flexible, wristed instruments with unlimited rotation, complex procedures can be performed. The patient‐side Roblens bedside camera integrates with the surgical bed, enabling uninterrupted bed reorientation during surgery and enhancing maneuverability, workflow efficiency, and suitability for procedures like colorectal surgery. Unique features include adjustable console handles to reduce surgeon fatigue and compatibility with existing laparoscopic vision systems, monitors, insufflators, and electrocautery equipment, which increases flexibility and reduces installation costs.

#### 3.2.9 Dexter

The Dexter Robotic System, developed in Switzerland, is an open robotic platform designed to optimize MIS and received CE mark approval in 2020 [5, 40]. It comprises a surgeon console, two patient‐side arms with 7 DoF and 75° angulation, and a robotic endoscope arm. Dexter offers instrument articulation, precision, and ergonomic flexibility with adjustable sitting or standing positions. The system is compatible with all laparoscopic instruments and towers, facilitates docking and instrument changes, and can switch to laparoscopic mode with a single command, allowing the surgeon to operate in a traditional laparoscopic setup without undocking.

#### 3.2.10 Bitrack System

The Bitrack system (Rob Surgical, Barcelona, Spain) is a teleoperated platform for MIS [41]. Developed in 2018, it has begun FDA/CE approval processes [42]. The system features a four‐arm robot on a column, with independently pivoting arms; the two upper arms use SCARA‐type architecture to avoid interference with lower arms [43].

An open console provides 3D glasses and ergonomic seating with an adjustable armrest. Instruments are 8‐mm, wristed, single‐use, with 7 DoF, supporting monopolar and bipolar energy. The system offers 3D‐HD vision [44] and incorporates artificial intelligence (AI) features, including respiratory compensation and Intelligent Laparoscopic Navigation. Compared with da Vinci, Bitrack is smaller, more cost‐efficient, and occupies less operating room space due to its single‐column design [45].

#### 3.2.11 Mantra

The Mantra robotic surgery system, developed by SS Innovations International (India), received approval from the Central Drugs Standard Control Organization (CDSCO) [44]. It features an open surgeon console with hand controls (mini joystick) [46, 47], foot pedal, and dual 3D/2D monitors. The patient cart includes 3–5 modular robotic arms, free from patient docking, with wristed, reusable instruments [41]. Mantra employs a 3D‐HD chip‐on‐tip articulating scope with 4 DoF, four‐way camera articulation, and head‐tracking safety [47]. While advanced energy instruments are under development, the system supports MIS across multiple specialties and multiport procedures. The console ergonomics allow adjustable seating for the surgeon.

#### 3.2.12 MicroHand S

The MicroHand S Surgical Robot, developed by Shandong WEGO Surgery Robot Co., Weihai, China, is a teleoperated master‐slave system for MIS [48]. It received NMPA approval in 2021 for select general surgery procedures. The patient cart includes three robotic arms—one for the endoscopic camera and two for instruments—mounted on a base [44].

The open surgeon console features an armrest, finger‐grip controllers, a stereo image viewer, dual master manipulators, a control panel, and foot pedals. MicroHand S offers advanced ultrasonic energy, is lightweight, cost‐effective, compatible with various equipment, and incorporates 5G technology for remote operations [49]. It has been applied across cardiothoracic, urology, general surgery, head and neck, and gynecology, with demonstrated effectiveness in procedures such as total mesorectal excision and sigmoidectomy, showing outcomes comparable to da Vinci Si [47].

#### 3.2.13 Saroa

The Saroa surgical robot employs pneumatic power, resulting in a compact, lightweight, and cost‐effective design compared with electrically powered systems like da Vinci [50]. Pneumatic actuation allows delicate, soft movements, reducing mechanical load on the patient and enabling assistants to work close to the robot.

The open surgeon console features a navigation monitor displaying quantitative and visual force feedback, enhancing surgical precision and collaboration. The instruments' gripping force is estimated and fed back to the operator for safer, more precise procedures. Saroa is compatible with endoscopes, monitors, and electrocautery equipment from various manufacturers, increasing flexibility and reducing installation costs. Overall, Saroa emphasizes safety, precision, and cost‐effectiveness through its pneumatic design and open‐platform compatibility [50].

#### 3.2.14 Toumai or Microport

Toumai, the first four‐arm robotic system in China, is used in over 30 centers and received NMPA approval for urology in 2022 [51]. Its setup resembles da Vinci Xi, with four arms mounted on a boom. The closed surgeon console features hand controls, a foot pedal clutch, and 3D‐HD vision. The system incorporates tremor filtration and 5G capability for remote operation, using 8.5‐mm instruments with 7 DoF.

The second‐generation Toumai is awaiting NMPA approval, and a single‐arm version is entering clinical trials. Specialized in urology, it supports multiport procedures and is applied in MISs such as prostatectomy and nephrectomy [47, 52].

### 3.3 Single Port Surgical Robotic Systems

#### 3.3.1 da Vinci SP

The da Vinci SP system enables minimally invasive surgery through a single incision or natural orifice. It comprises a patient cart with a single robotic arm, a surgeon console, and a vision cart. The robotic arm provides a 3D‐HD camera and three‐wristed instruments capable of articulation and triangulation. The surgeon console includes a relocation pedal and navigation interface to control the instrument [53]. SP has been applied in various urologic procedures, including prostatectomy, cystectomy, nephrectomy, and pyeloplasty, with positive outcomes reported across multiple institutions [54, 55, 56].

#### 3.3.2 Medrobotics Flex

The Medrobotics Flex System, developed by Medrobotics Corp., Raynham, MA, USA, received FDA clearance in July 2015 and CE mark approval in March 2016 [57]. It is the world's first steerable and shapeable robotic system for minimally invasive colorectal procedures in the United States.

Flex is a flexible endoscope system controlled by the surgeon, comprising four main components: the Flex console (with control handle and touch‐screen display), Flex base (converting electronic signals into mechanical motion), Flex Scope (single‐use, sterile endoscope with articulating segments), and Flex cart/stand. The system allows nonlinear maneuvering to reach targets within the pharynx and larynx and introduces flexible instruments along the path, which is advantageous for distally located lesions [58, 59].

The endoscope has concentric inner and outer segments; the distal segment, controlled via joystick, contains an HD digital camera, LED illumination, lens washer, and two accessory channels for 3.5‐mm instruments [60].

#### 3.3.3 MIRA

MIRA is a minimally invasive master‐slave device designed for surgical procedures in the United States [61]. Its miniaturized architecture allows two sterile, wristed, reusable arms and optics to enter the body through a single 2.5‐cm port. The compact main unit (~2 lbs) is portable and space‐efficient. Instruments feature articulating flex tips for precise manipulation [41].

The open surgeon console maintains peripheral vision and includes hand controls with open‐close paddles, clutch and camera functions, and foot pedals [51]. Real‐time Full HD display and a haptic indicator alert the surgeon when instruments are out of range.

MIRA has been applied in bowel resections, with the first right hemicolectomy reported in August 2021. Currently an investigational device, MIRA is not commercially available and is planned for deployment on the International Space Station under NASA funding [62].

#### 3.3.4 ENOS (SPORT)

The SPORT system, developed by Titan Medical (Toronto, Canada), is designed for SP surgery [62]. It deploys two articulated flexible instruments and two cameras (2D‐HD and 3D‐HD) through a 25‐mm insertion tube, with multi‐articulated arms allowing four‐quadrant access without external moving parts [44]. The system uses electrical actuation and an open surgeon console with hand controllers, foot pedal, and elbow rest [47].

Instruments are sterilizable and reusable, with 10 interchangeable tips, including monopolar hook, bipolar dissectors, needle driver, suture cutter, tenaculum, fenestrated, and laparoscopic clinch effectors. SPORT includes a seated, adjustable workstation and six arms integrated into the operating table, enabling precise control and flexibility during surgery. The system has been used in animal and human cadaver studies, with potential applications in cholecystectomy, fundoplication, and gynecology [41, 44, 47].

#### 3.3.5 SHURUI SP Robot

**Figure 1:**
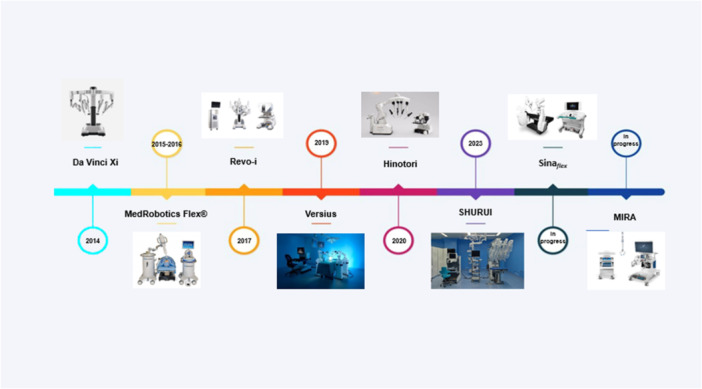
Examples of robotic surgery systems. This timeline illustrates the development of robotic surgery systems, from commercialized (the date when they received national or international clearance) to ongoing projects, featuring robots such as da Vinci Xi, Rev‐1, MedRobotics Flex, Versius, Hinotori, SHURUI, MIRA, and Sina_*flex*_.

The SHURUI system, developed by Beijing Surgeries Robotics Co., is designed for SP surgeries [62, 63, 64, 65]. It features a semi‐closed surgeon console with a 2D external display and a 3D high‐resolution monitor. The patient cart contains four independent arms, including a 3D endoscope and three deformable, reusable instruments introduced through a 25‐mm multichannel trocar. Each arm provides a large workspace and high maneuverability via an innovative dual continuum mechanism. SHURUI has completed clinical trials approved by the NMPA for urology and gynecology procedures in China [63, 64, 65, 66] (Figure 1).

## 4 Discussion

Based on the findings of this narrative review, telesurgery provides several advantages over conventional surgical approaches. Key benefits include eliminating the need for long‐distance patient travel, expanding access to care in underserved regions, facilitating remote surgical collaboration, enhancing surgical precision, helping address the global shortage of skilled surgeons, and reducing infection risk. Despite these strengths, telesurgery also faces notable limitations. Major barriers include limited high‐quality global network infrastructure, unresolved legal and regulatory issues, and the substantial costs of system acquisition, maintenance, and telesurgical procedures. Additional concerns involve vulnerability to cyberattacks, latency in data transmission between surgeon‐side and patient‐side systems, and the physical footprint of large robotic platforms, which may complicate operating room workflow. Moreover, telesurgical systems still carry a risk of organ injury due to occasional visualization and perception challenges [38].

An open and competitive market is expected to reduce costs, resolve existing challenges, and improve global access to robotic surgery. However, long‐term dominance of the da Vinci surgical system created a monopoly in the surgical robotics field, mainly due to the extensive patent protection held by Intuitive Surgical [6]. Following the expiration of several key patents in 2019, the market became accessible for new robotic systems to enter and compete [67]. Emerging robotic companies must carefully evaluate their marketing and pricing strategies to maintain competitiveness. Each newly developed robotic system introduces specific features aimed at overcoming the technical or financial limitations associated with the da Vinci platform. While some of these systems have already received approval for clinical use, others are currently in various stages of global deployment.

As major corporations incorporate advanced technologies into the development of surgical robots, growing market competition is expected to drive innovation and reduce overall costs for hospitals and end users [68]. The Revo‐I system reports a 42% reduction in procedural costs [30], while the Senhance platform demonstrates a 37% decrease [68]. The Avatera system further lowers expenses by using disposable instruments, eliminating sterilization requirements [69]. The Senhance robot also integrates standard laparoscopic tools—offering a more economical alternative to specialized robotic instruments—and has lower maintenance costs than the da Vinci system [67]. Similarly, the Sinaflex platform reports substantial reductions in system, maintenance, and per‐case consumable costs through affordable disposable instruments and has been specifically developed to meet the needs of healthcare systems in resource‐limited countries.

Although the initial investment and operational expenses of robotic systems are considered limiting factors, the overall cost‐effectiveness of robotic surgery remains controversial. Potential long‐term benefits—including reduced surgical complications, shorter recovery time, elimination of long‐distance travel, and improved surgeon ergonomics—may ultimately offset the upfront costs. For instance, the cost‐effectiveness of robotic distal pancreatectomy (RDP) compared with laparoscopic distal pancreatectomy (LDP) has been evaluated in recent studies [70, 71, 72]. Findings from the Spanish healthcare system indicate that RDP may offer greater cost‐effectiveness than LDP.

From another perspective, comprehensive training programs are essential for surgeons to gain proficiency in robotic‐assisted procedures, as each operation presents a distinct learning curve [73]. Regular assessment of surgeons' technical abilities is necessary to determine their position on the learning curve and to monitor skill progression and performance [74]. However, comparing learning curves across robotic systems is challenging due to multiple influencing factors, including robot design, procedural complexity, and prior surgical experience. Learning curves are typically evaluated using functional outcomes such as operative time, blood loss, lymph node yield, and length of hospital stay as surrogates for technical performance [73]. Notably, no correlation was found between total training hours and surgeon performance on the Sinaflex robotic telesurgery system [75]. Early evidence regarding the Hugo RAS platform suggests efficient setup and docking, with a learning curve comparable to other robotic systems [76]. Although specific data for the Versius system are limited, learning curves inherently vary based on the robotic platform, surgical specialty, and individual surgeon characteristics.

The integration of AI into robotic‐assisted surgery represents a major advancement in modern medicine. Contemporary robotic platforms operate as sophisticated human–machine systems that enable precise monitoring of surgical actions. AI can leverage this intraoperative data to enhance feedback to the surgeon, thereby improving surgical accuracy and clinical outcomes [77]. AI tools also support surgical decision‐making by assessing the need for acute surgical intervention, predicting the likelihood of procedural success, and forecasting postoperative complications with notable accuracy. Looking ahead, AI‐enhanced robotic systems are expected to transform surgical practice by tailoring operative strategies to individual patients, optimizing intraoperative workflows, and offering advanced educational tools for surgeons at all experience levels. Although a fully autonomous AI‐driven surgical robot replacing human surgeons is unlikely, AI is designed to augment—not substitute—surgeons' cognitive and technical capabilities [78].

Robotic surgery systems contribute to sustainability by incorporating reusable components, thereby reducing dependence on disposable instruments and lowering medical waste [79]. Additionally, robotic‐assisted procedures are associated with shorter hospital stays, fewer complications, and reduced morbidity, collectively diminishing the environmental footprint of healthcare delivery [80]. These improvements not only enhance patient outcomes but also decrease resource consumption linked to prolonged hospitalization. Energy efficiency is another important aspect of robotic system design, with current advancements aiming to reduce energy demand and improve overall sustainability. As AI becomes further integrated into robotic platforms, additional gains in operational efficiency and environmental responsibility are anticipated, positioning these systems as both technologically advanced and ecologically conscious.

In conclusion, robotic surgical systems—most notably the da Vinci platform—have markedly advanced abdominal surgery by expanding the capabilities of minimally invasive procedures. These systems offer distinct advantages and limitations, with their overall impact depending on the clinical context, institutional resources, and patient needs. Although high acquisition and maintenance costs remain significant barriers, ongoing technological innovation and increasing market competition are reshaping the field, enabling improved clinical outcomes and expanding access to robotic surgery, particularly in urology. Looking ahead, the trajectory of robotic surgery is directed toward greater precision, reduced invasiveness, and enhanced surgical efficiency. The integration of cutting‐edge technologies, especially AI, is expected to further support surgeons, streamline operative workflows, and elevate the overall safety and effectiveness of surgical care.

## Author Contributions

**Mashallah Torabi:** conceptualization, project administration. **Mehrnaz Aghanouri:** conceptualization, investigation, validation, writing – original draft, writing – review and editing. **Fatemeh Hadavandsiri:** conceptualization, investigation, methodology, validation, writing – original draft, writing – review and editing. **Maryam Goodarzi:** investigation. All authors have read and approved the final version of the manuscript. Fatemeh Hadavandsiri, the corresponding author, takes full responsibility for the integrity and accuracy of the manuscript.

## Conflicts of Interest

The authors declare no conflicts of interest.

## Transparency Statement

The lead author, Fatemeh Hadavandsiri, affirms that this manuscript is an honest, accurate, and transparent account of the study being reported; that no important aspects of the study have been omitted; and that any discrepancies from the study as planned (and, if relevant, registered) have been explained.

## Data Availability

Data sharing is not applicable to this article as no new data were created or analyzed in this study.
