# Correction to “Comprehensive Comparison of Features of Robotic Surgery Systems in Abdominal Surgeries: A Narrative Review Study”

**DOI:** 10.1002/hsr2.72589

**Published:** 2026-06-04

1

M. Torabi, M. Aghanouri, F. Hadavandsiri, and M. Goodarzi, “Comprehensive Comparison of Features of Robotic Surgery Systems in Abdominal Surgeries: A Narrative Review Study,” *Health Science Reports* 9 (2026): e71894. https://doi.org/10.1002/hsr2.71894.

The first author's affiliation has been added as Affiliation 1, resulting in the renumbering of the subsequent affiliations. The second author was previously listed with only one affiliation and has now been correctly associated with Affiliations 2 and 3.

The correct affiliations are as follows:

Mashallah Torabi: Research Center for Science and Technology in Medicine, Service Desk and Office Automation, Tehran University of Medical Sciences, Tehran, Iran.

Mehrnaz Aghanouri: Medical Image and Signal Processing Research Center, School of Advanced Technologies in Medicine, Isfahan University of Medical Sciences, Isfahan, Iran.

Department of Medical Physics and Biomedical Engineering, School of Medicine, Tehran University of Medical Sciences, Tehran, Iran.

The online version of this article has been corrected accordingly.

We apologize for this error.

